# Progressively Hybrid Transformer for Multi-Modal Vehicle Re-Identification

**DOI:** 10.3390/s23094206

**Published:** 2023-04-23

**Authors:** Wenjie Pan, Linhan Huang, Jianbao Liang, Lan Hong, Jianqing Zhu

**Affiliations:** 1 College of Engineering, Huaqiao University, Quanzhou 362021, China; 2 Xiamen Yealink Network Technology Company Limited, No. 666, Hu’an Road, High-Tech Park, Huli District, Xiamen 361015, China

**Keywords:** multi-modal image, transformer, vehicle re-identification

## Abstract

Multi-modal (i.e., visible, near-infrared, and thermal-infrared) vehicle re-identification has good potential to search vehicles of interest in low illumination. However, due to the fact that different modalities have varying imaging characteristics, a proper multi-modal complementary information fusion is crucial to multi-modal vehicle re-identification. For that, this paper proposes a progressively hybrid transformer (PHT). The PHT method consists of two aspects: random hybrid augmentation (RHA) and a feature hybrid mechanism (FHM). Regarding RHA, an image random cropper and a local region hybrider are designed. The image random cropper simultaneously crops multi-modal images of random positions, random numbers, random sizes, and random aspect ratios to generate local regions. The local region hybrider fuses the cropped regions to let regions of each modal bring local structural characteristics of all modalities, mitigating modal differences at the beginning of feature learning. Regarding the FHM, a modal-specific controller and a modal information embedding are designed to effectively fuse multi-modal information at the feature level. Experimental results show the proposed method wins the state-of-the-art method by a larger 2.7% mAP on RGBNT100 and a larger 6.6% mAP on RGBN300, demonstrating that the proposed method can learn multi-modal complementary information effectively.

## 1. Introduction

The aim of vehicle re-identification (ReID) [1,2,3] is to retrieve a specific vehicle image from a large-scale vehicle gallery captured by non-overlapping cameras, which receives a lot of attention from the artificial intelligence research field due to its significant role in intelligent transportation systems for building smart cities. Most existing vehicle ReID methods [4,5,6,7,8,9,10,11,12,13,14,15,16] are only based on single-modal visible images, i.e., RGB images, which would suffer from weak performance because of the poor imaging quality under low light environments.

**Figure 1:**
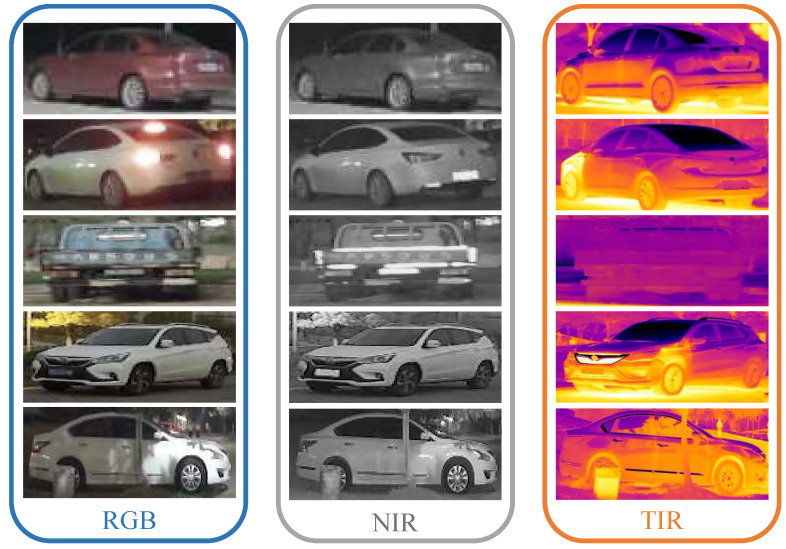
Multi-modal vehicle image examples. Here, RGB, NIR, and TIR are abbreviations for visible, near-infrared, and thermal-infrared, respectively.

To overcome low illumination conditions, Li et al. [17] firstly proposed using three-modal (i.e., visible, near-infrared, and thermal-infrared) images for vehicle ReID, and constructed a vehicle ReID benchmark that shows that three-modal vehicle ReID greatly improves accuracy in low illumination conditions. Although a non-visible spectrum could show good night imaging results to play good complements to visible images, different spectra have different imaging characteristics, which could be a challenge even to a strong global feature modeling model [16]. As shown in Figure 1, the contrast between the foreground (i.e., vehicles) and background in near-infrared images is lower than that in visible images. Visible images have a stronger ability to reflect texture detail information of vehicles than near-infrared images in the daytime. Thermal-infrared images contain more noise than visible and near-infrared images. As a result, although non-visible images have great potential to boost vehicle ReID performance in low illumination environments, there is an open question in multi-modal ReID in practice: how to effectively fuse the complementary information from multi-modal data?

Existing multi-modal vehicle Re-ID [17,18,19,20] most focus on learning modal robust features. For example, Wang et al. [20] designed a cross-modal interacting module and a relation-based embedding module to exchange useful information from multi-modal features so as to enhance features’ richness. Both cross-modal interacting and relation-based embedding modules are convolutional neural network (CNN) branches. Zheng et al. [19] proposed a cross-directional consistency network to mitigate cross-modal discrepancies and adjust individual feature distributions for learning modal robust features. Li et al. [17] proposed a heterogeneity collaboration aware multi-stream convolutional neural network to constrain scores of different instances of the same identity to be coherent. Guo et al. [21] proposed a generative and attentive fusion network to fuse and align features of the original data. Although they have acquired great progress for multi-modal vehicle ReID, there is still room for designing an effective multi-modal fusion manner to improve multi-modal vehicle ReID. Specifically, there are two reasons for emphasizing multi-modal fusion. First, current multi-modal vehicle ReID works [17,18,19,20,21,22] are based on CNNs that use local kernels having a limited receptive field, which is inadequate in fusing global features of multi-modal data. Hence, this paper designs a multi-modal hybrid transformer to use the transformer’s long-distance dependency learning ability to realize a global feature fusion of multi-modal data. Second, current multi-modal vehicle ReID methods only pay attention to the feature level fusion, and the image level fusion is underestimated. Therefore, this paper proposes a random hybrid augmentation to fuse multi-modal complementary information at the image level. Consequently, combing the multi-modal hybrid transformer and the random hybrid augmentation, a progressively hybrid transformer is constructed in this paper, which fuses multi-modal complementary information at both image and feature levels.

The contributions of this paper are summarized as follows:

- This paper proposes a multi-modal hybrid transformer, which applies the feature hybrid mechanism (FHM) to fuse multi-modal information at the feature level by the modal-specific controller and modal information embedding.
- This paper designs a random hybrid augmentation (RHA) to fuse multi-modal information at the image level, which upgrades the multi-modal hybrid transformer into a progressively hybrid transformer (PHT) that fuses multi-modal information at both image and feature levels.
- Experimental results on RGBNT100 and RGBN300 demonstrate that the proposed PHT outperforms state-of-the-art methods.

This paper is an extended version of the preliminary work [23]. Compared with the preliminary work [23], this paper is improved in two aspects. (1) This paper designs a new data augmentation method (i.e., random hybrid augmentation) to form a more comprehensive multi-modal information fusion which outperforms [23] a larger 0.9% mAP on RGBNT100 and a larger 0.3% mAP on RGBN300. (2) This paper implements more experiments to analyze the proposed method. The rest of this paper is organized as follows. Section 2 contains recent works related to the proposed method. Section 3 describes the proposed method in detail. Section 4 presents experimental results and analysis to show the proposed method’s advantage. Section 5 concludes this paper.

## 2. Related Works

### 2.1. Visible Re-Identification

Most of the existing vehicle re-identification methods are based on visible images and they have acquired great progress [6,8,9,24,25,26,27,28]. Several representative works are reviewed as follows. Zhu et al. [5] extracted the final similarity by using orientation and camera similarity as auxiliaries to alleviate the difficulty of similar appearances. Cai et al. [29] proposed a multi-level feature extracting approach to learn global features from whole vehicle images and learn local discriminative features from different local region channels. Meng et al. [7] proposed a part perspective transformation module to map the different vehicle parts into a unified perspective to deal with viewpoint variations. Zhou et al. [8] proposed a viewpoint-aware attentive multi-view inference model cooperating with visual information to handle viewpoint variations. Li et al. [27] proposed an efficient transformer to learn multi-view part-wise correlations to deal with complex viewpoint variations. Zeng et al. [30] proposed an illumination identity disentanglement (IID) network to dispel different scales of illumination away while maintaining each identity’s discriminant information. Zhang et al. [31] proposed using an illumination teacher model trained by the differences between the illumination-adjusted and original images to separate the ReID features from lighting features to enhance ReID performance. Although low illumination promotes vehicle ReID, extremely unsatisfactory illumination conditions are still killers of vehicle ReID.

### 2.2. Deep Architecture

Thanks to the rapid development of deep learning, many excellent deep networks have emerged in computer vision research fields, which could be divided into two categories: (1) convolutional-based networks [32,33,34,35,36,37,38,39,40] and (2) vision transformer-based networks [41,42,43,44,45,46,47,48,49].

The first convolutional neural network (CNN) is proposed by LeCun [32], which shows an impressive performance for document recognition. Krizhevsky et al. [33] proposed the famous AlexNet via stacking more convolutional layers followed by max-pooling layers and fully connected layers, acquiring good results on the large-scale image classification benchmark [50]. Simonyan et al. [34] emphasized using more small convolutional kernels to construct a deeper VGG network. Szegedy et al. [35] first designed GoogLeNet with an inception structure utilizing sparse structure to achieve deep and wide networks. Ioffe et al. [36] designed a batch normalization layer playing in a convolution layer and an activation function to reduce internal covariate shifts to improve the training convergence of GoogLeNet. Furthermore, Szegedy et al. [37] explored factorizing convolutions with large kernels to avoid representational bottlenecks of inception structures of GoogLeNet. In addition to inception series, residual networks [39,51,52] are another popular family. He et al. [39] firstly designed residual layers to effectively alleviate the problem of gradient vanishing, allowing for training ultra-deep networks, namely, residual networks (ResNet). Hu et al. [52] designed a squeeze-and-excitation (SE) block to learn channel-wise information to upgrade the ResNet to the SE-ResNet. Xie et al. [51] proposed ResNeXt by combining the residual layer and the inception structure. Szegedy et al. [38] also combined the inception structure and the residual layer to improve their networks.

More recently, vision transformer [49], known for its ability to learn global features from its self-attention mechanism, has done an impressive job in computer vision tasks. Wu et al. [53] proposed a pyramid pooling method to acquire a stronger multi-head self-attention that could more properly deal with multi-scale information. Zhang et al. [24] introduced a transformer-based feature calibration to integrate low-level detail information as a global prior for high-level semantic information. Chen et al. [54] proposed a structure-aware positional transformer network to utilize the structural and positional information and learn semantic-aware features. Especially, for the visible modal person/vehicle ReID task, He et al. [16] first proposed a pure transformer-based object ReID framework, which achieves state-of-the-art performance on most person/vehicle re-identification benchmarks.

### 2.3. Data Augmentation

Zhong et al. [55] proposed a data augmentation method to randomly select a rectangle region in an image and erase its pixel with a random value, which reduces the risk of over-fitting and makes a deep network robust to occlusions. The random patch method [56] firstly creates a patch pool of random image patches and then pastes a random patch from the patch pool onto an input image at a random position. Because [55,56] could heavily occlude images, Chen et al. [57] believed these two methods would harm the models’ ability to mine salient local information, so they proposed soft random erasing, in which an erased area is not completely replaced with random pixels but also retains a proportion of the original pixels. Li et al. [58] combined different regions of different identities to generate virtual regional perceptual data pairs. Qjagh et al. [59] proposed a data preprocessing strategy to generate the missing data by average, maximum, and weighted average. Lin et al. [60] proposed an illuminate-aware data-augmentation method that estimates the illuminate distribution from the training data and generates synthesis images under different illumination. Huang et al. [61] designed an adversarial learning-based occlusion image generation method to enhance the person ReID model’s generalization ability.

Considering these data augmentation methods perform well by introducing useful complementary information and the complementary information between different modalities is essential for multi-modal vehicle ReID, a random hybrid augmentation (RHA) method is designed to improve the previous work [23] in the fusion of the image level. Compared with the previous work, [23], which only fuses multi-modal information at the feature level, this paper fuses multi-modal information at both image and feature levels. Specifically, in addition to the multi-modal information fusion at the feature level, this paper fuses multi-modal information at the image level by exchanging information between different modalities at image regions with random positions, random numbers, random sizes, and random aspect ratios.

## 3. Methodology

**Figure 2:**
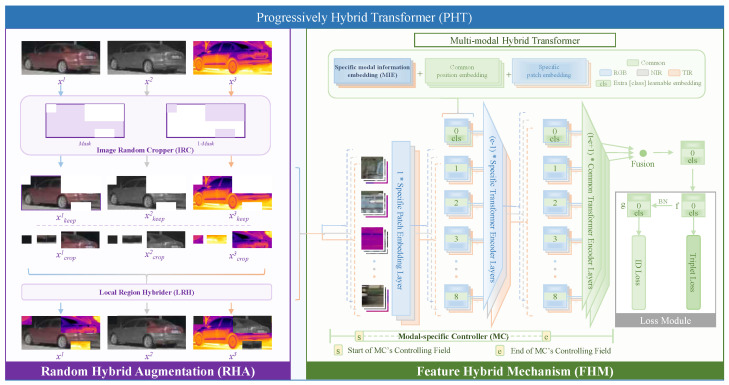
The overall framework of the proposed progressively hybrid transformer.

Figure 2 shows the overall framework of the proposed progressively hybrid transformer (PHT), including (1) random hybrid augmentation (RHA) and (2) a feature hybrid mechanism (FHM)-based multi-modal hybrid transformer. RHA brings local structural characteristics of all modalities, mitigating modal differences at the beginning of feature learning. The FHM assigns the distribution of modal-specific layers to improve multi-modal feature fusion.

### 3.1. Random Hybrid Augmentation

As shown in Figure 2, the RHA has two processors: (1) a image random cropper (IRC) and (2) a local region hybrider (LRH). The IRC extracts multi-modal-specific information by simultaneously cropping multi-modal images of random positions, random numbers, and random sizes. The LRH captures multi-modal complementary information by fusing the cropped regions to let regions of each modal take local structural characteristics of multi-modalities.

Given a group of *n*-modal images $\left\{x^{i}\in R^{H\times W},i=1,2,\ldots ,n\right\}$, where *H* and *W* denote the height and width of each modal image. For the convenience of description, the IRC is parameterized by $n_{region}$ and $p_{region}$, which, respectively, denotes the max number of cropped regions and the max proportion of the cropped edge and the image original edge. As shown in Figure 2, the IRC’s workflow is described as follows.

(1) Initializing a H×W sized Mask whose elements are equal to 1.
(2) Random zero setting $l\in [0,n_{region}]$ local regions of Mask, that is,
  (1) $$Mask\left(m,n\right)=\left\{\begin{array}{cc} 0 & m,n\in \cup_{j=1}^{l}R_{j},\\ 1 & \mathrm{otherwise}, \end{array}\right.$$
  where m∈[1,H] and n∈[1,W] are y-coordinate and x-coordinate, respectively; $R_{j}$ is the *j*-th zero setting region that has a random aspect ratio and a random area. Please note that each zero setting region’s max height and width are $H\times p_{region}$ and $W\times p_{region}$.
(3) Cropping each modal image as follows.
  (2) $$\begin{array}{c} x_{crop}^{i}=x^{i}⊗\left(1-Mask\right),\\ x_{keep}^{i}=x^{i}⊗Mask,\\ i=1,2,...,n, \end{array}$$
  where ⊗ is element-wise multiplication operation; $x_{crop}^{i}$ is the cropped part of the *i*-th modal image, and $x_{keep}^{i}$ is the rest part that keeps unchanging.

Based on Equation (2), the LRH calculation is formulated as follows:

(3) $$x^{i}=x_{keep}^{i}+Hybrid\left(x_{crop}^{1},x_{crop}^{2},...,x_{crop}^{n}\right),$$

where Hybrid is the fusion function. In this paper, five types of fusion functions are designed. (1) The average method, which simply averages all modal cropped regions. (2) The self-excluding average, which first excludes cropped regions of its own modality and then averages cropped regions of all remaining modalities. Similarly, two Hadamard product versions are also designed, i.e., (3) the Hadamard product and (4) the self-excluding Hadamard product. (5) Randomly swapping, in which $\left\{x_{crop}^{1},x_{crop}^{2},...,x_{crop}^{n}\right\}$ are stochastically scheduled and then each element is used to replace the cropped regions of a modality. Based on Equations (2) and (3), the RHA module could bring local structural characteristics of all modalities, reducing modal differences at the beginning of feature learning.

### 3.2. Feature Hybrid Mechanism-Based Multi-Modal Hybrid Transformer

As shown in Figure 2, this paper designs a multi-modal hybrid transformer, which is a multi-branch transformer simultaneously extracting features from multi-modal images. Each branch is a vision transformer proposed by [16,42], which consists of a patch embedding layer and a list of encoders. The patch embedding layer is responsible for mapping the image patch into a vector. The encoder is a combating of layer normalization and multi-head self-attention with residual connections to complex features of vectors generated by the patch embedding layer. Features from each branch are fused to form multi-modal features and are fed into the loss function for training. In this paper, three feature fusion methods are applied, i.e., (1) average, (2) Hadamard product, and (3) concatenation.

The multi-modal hybrid transformer only fuses multi-modal information at one and only one depth position. Hence, the feature hybrid mechanism (FHM) is proposed to improve the multi-modal hybrid transformer. The FHM has two modules: (1) modal-specific controller (MC), and (2) modal information embedding (MIE). The MC module is designed for allocating the modal-specific parts of vision transformer branches. The MIE module is designed to attach modal information to patch embeddings. The details of the MC and MIE are described as follows.

#### 3.2.1. Modal-Specific Controller

The MC module assigns the sharing attribute of three structures, i.e., (1) position embedding, (2) patch embedding layers, and (3) encoders. For the position embedding, the MC module default set the position embedding to be modal-common, considering that spatial position information is more likely to be modal independent.

For patch embedding layers and encoders, the MC module can flexibly assign common or specific attributes with a modal-specific controlling field and the number of modal-specific layers. The modal-specific controlling field is denoted as v=[s,e), where *s* and *e* are natural numbers, and the number of modal-specific layers is written as *k*, where k≤e−s. Given a transformer model of one patch embedding layer and *t* encoders, the MC workflow is formulated in Equation (4).

(4) $$MC\left(k,s,e,i\right)=\left\{\begin{array}{cc} \mathrm{modal}-\mathrm{specific}, & i\in [s,e)\cap [s,s+k),\\ \mathrm{modal}-\mathrm{common}, & \mathrm{otherwise}, \end{array}\right.$$

where i∈[0,t+1) represents the transformer component index, and the patch embedding layer index is i=0. Through Equation (4) of the MC module, the first *s* layers are modal common, the next *k* layers are modal specific, and the last t+1−k layers are modal common.

Figure 2 shows the case that has s=0,e=t,k=e−s. For example, as a transformer model has 12 encoders, in the medium modal-specific configuration of k=9,v=[1,10), the patch embedding layer is modal common, the first 9 encoders layers are modal specific, and the rest of the three encoders layers are modal common.

#### 3.2.2. Modal Information Embedding

Different from the position embedding, *P* is set as the modal-common default, the modal information embedding MIE is always set as modal specific to freely encode modal information to alleviate the feature deviations towards modal variations. Inspired by [41], the modal information embedding is formulated in Equation (5), as follows:

(5) $$Z=[x_{cls};E\left(x_{p}^{1}\right);E\left(x_{p}^{2}\right);\ldots ;E\left(x_{p}^{N}\right)]+P+MIE,$$

where *Z* denotes the output of patch-embedding layers (i.e., E(·)); $x_{cls}$ is a learnable token embedding; $x_{p}$ is a image patch, and *N* is the number of patches; *P* is a learnable position embedding; MIE is a learnable modal information embedding.

### 3.3. Progressively Hybrid Transformer

Combining the proposed RHA and FHM designed in previous subsections, the multi-modal hybrid transformer would be upgraded into a progressively hybrid transformer (PHT) because both image and feature level information is progressively fused. As shown in Figure 2, the PHT’s loss module consists of a triplet loss and a classification loss. The triplet loss is the hard-miming triplet loss function [62] formulated in Equation (6), as follows:

(6) $$L_{tri}=log[1+exp(∥f_{a}-f_{hp}{∥}^{2}-∥f_{a}-f_{hn}{∥}^{2})],$$

where $f_{a}$ is the multi-modal fusion feature of an anchor sample, $f_{hp}$ is the multi-modal fusion feature of a hard positive sample that is the farthest away from the anchor sample and has the same class as the anchor sample, and $f_{hn}$ is the multi-modal fusion feature of a hard negative sample that is close to the anchor sample and has a different class from the anchor sample. The classification loss is the commonly used cross-entropy loss function [16] formulated in Equation (7), as follows:

(7) $$L_{cls}=-\delta \left(y==c\right)log\left(p\left(y|g\right)\right),$$

where δ is an indicator function that is equal to 1 if the equation in the formula is true, otherwise 0, *g* is the batch normalized multi-modal fusion feature of a sample, and *y* and *c* are the sample’s prediction and truth class labels, respectively.

## 4. Experiments and Analysis

To show the proposed method’s advantage, this paper compares the PHT method with state-of-the-art methods on two challenging multi-modal vehicle datasets, namely, RGBNT100 [17] and RGBN300 [17]. The RGBNT100 is a three-modal dataset, including visible, near-infrared, and thermal images of 100 subjects, and the RGBN300 is a two-modal dataset, containing visible and near-infrared images of 300 subjects. Following [17], on both RGBNT100 and RGBN300 datasets, half of the dataset is used for training and the other half is for testing. The cumulative matching characteristic (CMC) curve [63] and the mean average precision (mAP) [64] are applied as the performance metric. R1, R5, and R10 denote rank-1, rank-5, and rank-10 identification rates on a CMC curve, respectively.

### 4.1. Implementation Details

The software tools are Pytorch 1.7 [65], CUDA 11.1, and python 3.8. The hardware device is one GeForce RTX 3090 GPU. All images of each modality are resized to 192×192 sized images. The random horizontal flipping, padding, random cropping, and random erasing [55] are applied for data augmentation, as performed in [16]. Each mini-batch contains 16 subjects, and if on the RGBNT100 dataset, each subject has 4 visible images, 4 near-infrared images, and 4 thermal images, otherwise, on the RGBN300 dataset, each subject has 4 visible images and 4 near-infrared images. The ImageNet pre-trained vision transformer (ViT) is applied as the backbone as performed in [16]. Following [16], the momentum and weight decay of the stochastic gradient descent (SGD) optimizer [33] are set to 0.9 and 0.0001, respectively, the learning rate is initialized as 0.008 with cosine learning rate decay, and the patch size and stride size are both set to 16×16. As RGBNT100 and RGBN300 are three-modal and double-modal datasets, the PHT’s backbone is correspondingly made to have three ViT branches and two ViT branches on the RGBNT100 and RGBN300. As each ViT branch has 1 patch embedding layer and 12 transformer encoder layers, the controlled field of the modal-specific controller (MC) is limited to v=[s,e)|0≤s≤e≤13.

### 4.2. Comparison with State-of-the-Art

The performance comparison between the proposed PHT and state-of-the-art methods is shown in Table 1. Those state-of-the-art methods could be divided into two categories: (1) CNN-based methods, namely, HAMNet [17], GAFNet [21], CCNet [19], and DANet [22]; (2) the transformer-based method, namely, TransReID [16]. Several interesting observations are as follows.

First, the transformer-based method TransReID [16] is inferior to those CNN-based methods. For example, the mAP of TransReID [16] is 5.3% smaller than the earliest CNN-based method called HAMNet [17]. This observation illustrates that without an appropriate multi-modal information fusion, even using a strong transformer, there is no accuracy performance advantage.

Second, the proposed method (i.e., PHT) greatly improves TransReID [16] and outperforms those CNN-based methods. On RGBNT100, the PHT’s mAP is 1.8% larger than that of the strongest CNN-based method, i.e., CCNet [19], although R1, R5, and R10 of the PHT are inferior to those of CCNet [19]. According to [64], mAP is a more comprehensive performance indicator than R1, R5, and R10, who are isolated points on a CMC curve. Therefore, the PHT is better overall than CCNet [19]. Similarly, on RGBN300, the PHT gains good performance, which defeats the strongest one (i.e., GAFNet [21]) by a 6.6% larger mAP. These results suggest that the full fusion working at both image and feature levels is a great help for a transformer model to improve multi-modal vehicle ReID.

### 4.3. Analysis of Feature Hybrid Mechanism

#### 4.3.1. Influence of Modal-Specific Controller

**Figure 3:**
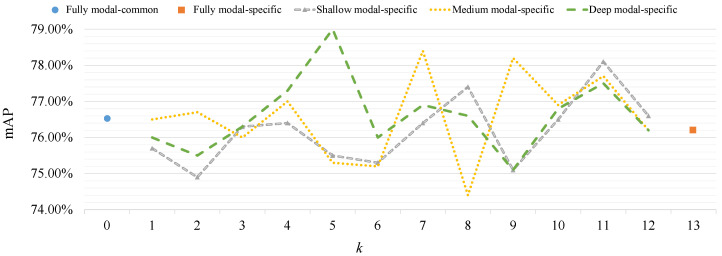
The comparison of modal-specific controller configurations on RGBNT100.

To investigate the influence of using modal-specific layers at different positions, five types of modal-specific controller (MC) configurations are formed based on Equation (4), as shown in Table 2. These configurations of the MC are conducted on RGBNT100. Furthermore, position embedding is set to be modal-common and disabled RHA to avoid their influence. The experimental results are shown in Figure 3.

From Figure 3 one can see that three partial modal-specific (i.e., shallow modal-specific, medium modal-specific, and deep modal-specific) configurations outperform fully modal-specific and fully modal-common configurations. Especially, when the deep modal-specific configuration has the number of modal-specific layers k=5 and controlled field v=[8,13), the best performance (79.0% mAP) is achieved. Furthermore, among three partial modal-specific configurations, the deep modal-specific configuration outperforms shallow modal-specific and medium modal-specific configurations. The strength of the deep modal-specific configuration setting shallow layers of a transformer to be modal-common is that the fusion computation works on a deep location requiring complementary features of different modalities so that modal-common layers should be configured at shallow positions while modal-specific layers should be configured at deep positions near to the fusion computation for fusing multi-modal complementary information better.

#### 4.3.2. Role of Modal Information Embedding

Based on the observation on the analysis of the modal-specific controller (MC) in Section 4.3.1, each type’s best MC configuration is chosen and RHA is discarded, and then the role of modal information embedding (MIE) is analyzed, as follows.

**Figure 4:**
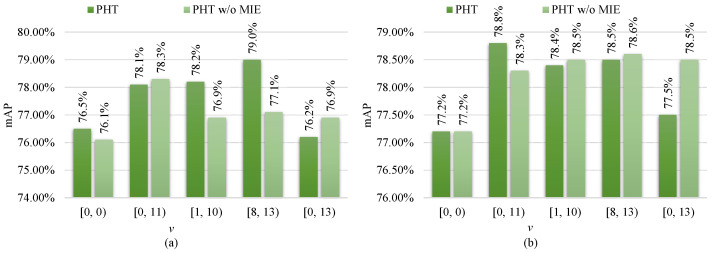
The ablation study of modal information embedding (MIE) on (**a**) RGBNT100 and (**b**) RGBN300 datasets. Here, *k* is configured to k=e−s.

From Figure 4, one can see that PHT with MIE outperforms the PHT without MIE by a 1.9% larger mAP on RGBNT100 and a larger 0.5% mAP on RGBN300, respectively, under the modal-specific configuration of v=[8,13). Unfortunately, using MIE brings a negative impact on RGBNT100 and RGBN300 under the fully modal-specific configuration of v=[0,13). This is because the fully modal-specific configuration has no modal-common layers, hindering MIE from learning modal invariant characteristics. Consequently, MIE is useful to alleviate feature deviations towards modal variations and is helpful to enhance multi-modal complementary information fusing but requires a proper MC configuration.

#### 4.3.3. Impact of Position Embedding

Similar to the experiment settings in the previous model information embedding (MIE) analysis, each type’s best MC configuration is chosen and RHA is discarded, and then the performance resulting from modal-specific and modal-common position embedding on RGBNT100 and RGBN300 is compared.

From Table 3, one can find that most modal-common position embedding cases are stronger than modal-specific position embedding. For example, on RGBNT100, regarding the v=[1,10) case, the mAP of modal-common position embedding is 1.5% larger than that of the modal-specific position embedding. Similarly, for the v=[8,13) case, the modal-common position embedding outperforms the modal-specific position embedding by a 1.4% mAP improvement. These results mean that the modal-common position embedding is more robust than the modal-specific position embedding. The reason for this situation is deduced to the modal-common position embedding requiring fewer parameters than the modal-specific position embedding so that it is easier to be well trained.

#### 4.3.4. Effect of Feature Fusion

According to Figure 3, the best configuration (i.e., k=5 and v=[8,13) in deep modal specific) are selected to compare the average, Hadamard product [66], and concatenating fusion methods. Here, the modal-common position embedding is applied and RHA is still disabled.

From Table 4, one can observe that the average fusion method gains the best result, that is, 79.0% mAP, 93.4% R1, 94.4% R5, and 95.3% R10 on RGBNT100, and 78.5% mAP, 92.3% R1, 93.1% R5, and 93.7% R10 on RGBN300. The preponderance of the average fusion method suggests that the low-pass effect of average fusion could filter out multi-modal heterogeneity of multi-modal data, so as to improve performance more significantly.

### 4.4. Analysis of Random Hybrid Augmentation

#### 4.4.1. Comparison with the Preliminary Work

**Figure 5:**
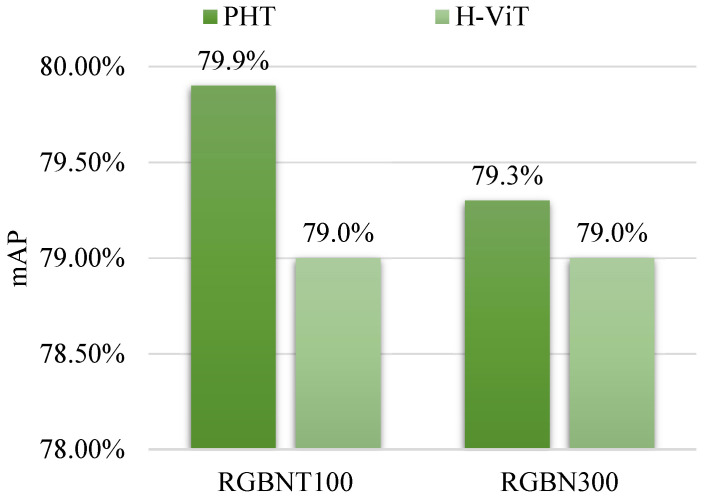
The comparison between PHT and the previous work H-ViT on RGBNT100 and RGBN300 datasets.

To straightforwardly show the role of random hybrid augmentation (RHA), this paper compares the proposed PHT to the preliminary work [23], namely, H-ViT, which does not utilize RHA. As shown in Figure 5, the PHT in this paper consistently outperforms H-ViT [23] on both RGBNT100 and RGBN300. This comparison illustrates that the fusion at the image level of RHA supplements the fusion at the feature level, further boosting multi-modal vehicle ReID. More detailed analyses of RHA are constructed as follows.

#### 4.4.2. Role of Image Random Cropper

**Figure 6:**
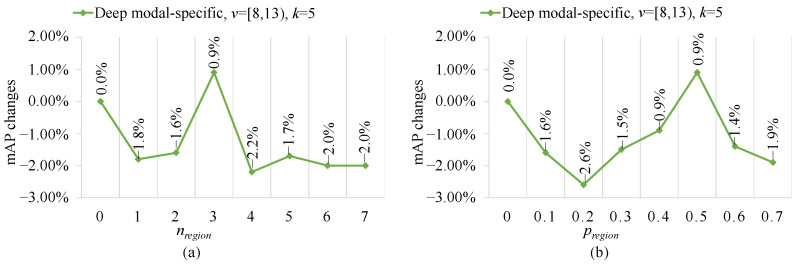
The role of parameters (**a**) $n_{region}$ and (**b**) $p_{region}$ in image random cropper on RGBNT100.

According to Figure 3, the best MC configuration (i.e., the deep modal-specific configuration of v=[8,13)) is fixed and two key parameters of the image random cropper (IRC), i.e., $n_{region}$ and $p_{region}$, are changed to validate the role of IRC. The results are shown in Figure 6a,b.

As shown in Figure 6a, one can see that the best $n_{region}$ value is 3 which brings 0.9% mAP performance improvements but most of the rest of the values cause performance degradation. This paper believes this small $n_{region}$ could not bring data augmentation while a too dominant $n_{region}$ could damage the original image information. Based on a similar reason, as shown in Figure 6b, the $p_{region}$ has a similar performance fluctuation trend, that is, performance improvements followed by performance degradation. Therefore, good RHA should have proper $n_{region}$ and $p_{region}$ settings for better multi-modal complementary information learning, as performed in existing data augmentation works [55,56].

#### 4.4.3. Impact of Local Region Hybrider

Based on observations in the previous subsection (i.e., Section 4.4.2) of IRC analysis, the impact of the local region hybrider is further analyzed by using different hybrid methods, including (1) average, (2) self-excluding average, (3) Hadamard product, (4) self-excluding Hadamard product, and (5) randomly swapping. The results are shown in Table 5.

From Table 5, it can be found that average reaches the best performance, i.e., 79.9% mAP, which defeats self-excluding average, Hadamard product, self-excluding Hadamard product, and randomly swapping by a 1.2%, 1.1%, 3.1%, and 1.9% mAP, respectively. This result is in line with the average preponderance of the average fusion method in the feature hybrid mechanism (i.e., Section 4.3.4), which demonstrates that the low-pass effect of average fusion could filter out multi-modal heterogeneity of multi-modal data again to improve performance more significantly.

### 4.5. Discussion

Based on the comparison with state-of-the-art methods in Section 4.2, the performance strength of the PHT is demonstrated. Specifically, the proposed PHT method is superior to the transformer-based method TransReID [16] by 19.8% mAP on RGBNT100 [17] and 12.2% mAP on RGBN300 [17]. Compared to two strong CNN-based methods, namely, GAFNet [21] and CCNet [19], the proposed PHT method outperforms GAFNet [21] by 2.7% mAP on RGBNT100 [17] and CCNet [19] by 6.6% mAP on RGBN300 [17]. Furthermore, based on ablation experiments in Section 4.3 and Section 4.4, the performance advantage of the PHT is demonstrated. Especially, compared to the preliminary work H-ViT [23], the proposed PHT mAP is 0.9% larger on RGBNT100 [17]. The victory of the proposed PHT in this paper demonstrates that image level information fusion is beneficial to feature level information fusion. The victory is actually expected because the fusion at the image level could be seen as a data augmentation, which is naturally conducive to the subsequent feature learning.

## 5. Conclusions

To comprehensively fuse multi-modal complementary information for multi-modal vehicle ReID, this paper proposes a progressively hybrid transformer (PHT). The PHT is constructed with two aspects: random hybrid augmentation (RHA) and a feature hybrid mechanism (FHM). At the image level, the RHA emphasizes structural characteristics of all modalities by fusing random regions of multi-modal images. At the feature level, the FHM allows for a multi-modal feature interaction by encoding modal information and fusing different modal features in different positions. The experiments show that (1) the proposed PHT surpasses the state-of-the-art methods on both RGBNT100 and RGBN300 datasets; (2) the multi-modal hybrid transformer built on the FHM is more advantageous than the single-branch transformer; (3) the fusion at the image level of RHA supplements the fusion at the feature level to further boost multi-modal vehicle ReID. Although the PHT is effective for multi-modal vehicle ReID, there is still a limitation of the PHT because it requires a manual setting of fusion configurations (e.g., fusion locations and fusion manners). In the future, a network architecture search approach will be explored to automatically determine fusion locations and manners to realize an adaptive fusion for multi-modal vehicle ReID.

## Figures and Tables

**Table 1:** The performance comparison between the proposed PHT and other state-of-the-arts methods on both RGBNT100 and RGBN300.

	RGBNT100				RGBN300			
Methods	mAP (%)	R1 (%)	R5 (%)	R10 (%)	mAP (%)	R1 (%)	R5 (%)	R10 (%)
HAMNet [17]	65.4	85.5	87.9	88.8	61.9	84.0	86.0	87.0
TransReID [16]	60.1	82.2	83.7	84.7	67.1	86.5	88.0	88.7
GAFNet [21]	74.4	93.4	94.5	95.0	72.7	91.9	93.6	94.2
CCNet [19]	77.2	96.3	97.2	97.7	N/A	N/A	N/A	N/A
DANet [22]	N/A	N/A	N/A	N/A	71.0	89.9	90.9	91.5
PHT (Proposed)	79.9	92.7	93.2	93.7	79.3	93.7	94.8	95.3

**Table 2:** Five types of modal-specific controller (MC) configurations.

Type	*v*		*k*	Patch Embedding Layer	Transformer Encoder Layers
	s	e			
Fully modal common	0	0	0	Common	Common: all Layers
Fully modal specific	0	t+1	t+1	Specific	Specific: all Layers
Shallow modal specific	0	*t*	1≤k≤t	Specific	Specific: the first k−1 layers
Medium modal specific	1	t+1	1≤k≤t	Common	Specific: the first *k* layers
Deep modal specific	1	t+1	1≤k≤t	Common	Specific: the last *k* layers

**Table 3:** The comparison of the modal-specific position embedding and the modal-common position embedding on RGBNT100 and RGBN300.

*v*	*k*	Type	RGBNT100				RGBN300			
			mAP (%)	R1 (%)	R5 (%)	R10 (%)	mAP (%)	R1 (%)	R5 (%)	R10 (%)
[0, 0)	0	Common	76.5	91.5	93.1	93.6	77.2	91.2	92.5	93.1
		Specific	76.1	91.5	92.9	93.4	77.8	92.8	93.6	93.8
[0, 11)	11	Common	78.1	91.9	92.7	93.2	78.8	93.5	94.5	95.2
		Specific	77.7	92.1	92.9	93.7	79.0	93.7	94.7	95.1
[1, 10)	9	Common	78.2	93.4	94.2	94.8	78.4	93.4	94.4	94.8
		Specific	76.7	91.7	93.1	93.9	78.4	93.2	94.2	94.8
[8, 13)	5	Common	79.0	93.4	94.4	95.3	78.5	92.3	93.1	93.7
		Specific	77.6	90.6	91.6	92.1	78.4	92.8	93.7	94.2
[0, 13)	13	Common	76.2	92.7	93.6	94.3	77.5	92.4	93.3	94.0
		Specific	76.9	92.8	94.2	94.6	77.2	92.5	93.2	93.7

**Table 4:** Results of different fusion methods on RGBNT100 and RGBN300.

Fusion	RGBNT100				RGBN300			
	mAP (%)	R1 (%)	R5 (%)	R10 (%)	mAP (%)	R1 (%)	R5 (%)	R10 (%)
Average	79.0	93.4	94.4	95.3	78.5	92.3	93.1	93.7
Hadamard Product	45.2	63.0	65.9	67.6	72.0	89.1	90.5	91.2
Concatenating	74.9	92.4	93.5	94.1	75.6	91.2	92.3	92.9

**Table 5:** Results of different local region hybrider on RGBNT100.

Local Region Hybrider	mAP (%)	R1 (%)	R5 (%)	R10 (%)
Average	79.9	92.7	93.2	93.7
Self-excluding Average	78.7	91.8	92.6	93.1
Hadamard Product	78.8	91.7	92.9	93.6
Self-excluding Hadamard Product	76.8	91.1	92.1	92.5
Randomly Swapping	78.0	91.0	92.1	92.7

## Data Availability

Not applicable.

## Abbreviations

The following abbreviations are used in this manuscript:

ReID	Re-identification
PHT	Progressively hybrid transformer
RHA	Random hybrid augmentation
FHM	Feature hybrid mechanism
NIR	Near-infrared
TIR	Thermal-infrared
CNN	Convolutional neural networks
IRC	Image random cropper
LRH	Local region hybrider
MC	Modal-specific controller
MIE	Modal information embedding
CMC	Cumulative matching characteristic
mAP	Mean average precision
R1	Rank 1 identification rate
R5	Rank 5 identification rate
R10	Rank 10 identification rate
ViT	Vision transformer
SGD	Stochastic gradient descent
